# Assessing mid-career female physician burnout in the military health system: finding joy in practice after the COVID-19 pandemic

**DOI:** 10.1186/s12889-024-18357-5

**Published:** 2024-03-20

**Authors:** Jessica Korona-Bailey, Miranda Lynn Janvrin, Lisa Shaw, Tracey Perez Koehlmoos

**Affiliations:** 1 Uniformed Services University of the Health Sciences, 4301 Jones Bridge Road, Bethesda, MD 20814 USA; 2 Henry M. Jackson Foundation for the Advancement of Military Medicine, 6720A Rockledge Dr, Bethesda, MD 20817 USA

**Keywords:** Physician burnout, Morale, Military medicine, Mental health, Female physicians

## Abstract

**Background:**

Rates of physician burnout increased during the COVID-19 pandemic and are expected to continue to rise. Mid-career physicians, female physicians, and military physicians have all been identified as potentially vulnerable populations to experience burnout. We examine factors associated with physician burnout among this intersectional group through a qualitative key informant interview study.

**Methods:**

We developed a semi-structured interview guide using the Institute for Healthcare Improvement’s Improving Joy in Work Framework and recruited military, mid-career female physicians who worked in the Military Health System(MHS) during the COVID-19 pandemic, (March 2020 -December 2021). Notes were collated and deductive thematic analysis was conducted.

**Results:**

We interviewed a total of 22 mid-career female physician participants. Participants were between 30 and 44 years of age and 7 were mothers during the pandemic. Most were White and served in the Army. All participants discussed the importance of building rapport and having a good relationship with coworkers. All participants also described their discontentment with the new MHS GENESIS electronic health record system. An emerging theme was military pride as most participants were proud to serve in and support the military population. Additionally, participants discussed the negative impact from not feeling supported and not feeling heard by leadership.

**Conclusions:**

Much like providers in other health systems during the pandemic, MHS physicians experienced burnout. This study allowed us to gather key insights to improve policies for active duty service mid-career female military physicians. Provider inclusion, autonomy, and work culture play critical roles in future systems improvement and workforce retention.

**Supplementary Information:**

The online version contains supplementary material available at 10.1186/s12889-024-18357-5.

## Introduction

Physician burnout is a mental health condition characterized by increased mental and emotional exhaustion, increased depersonalization, reduced sense of personal accomplishment, increased absenteeism, and decreased work productivity that results in worsened health outcomes for patients [1]. Approximately half of all physicians and residents experience burnout at some point in their career, more than twice the rate experienced by those in other similarly demanding positions [1]. Left unchecked, physician burnout can result in worsened quality of care, increased costs, and lack of access for patients [2]. Additionally, burnout is a contributing factor of physicians leaving the workforce spontaneously and early in their careers, which exacerbates ongoing physician shortages.

A review of the literature found that younger age, female sex, being unmarried, long work hours, and low reported job satisfaction were predictive of physician burnout [3]. Mid-career physicians were at increased risk, as they worked more hours compared to early and late career physicians, took more overnight calls, had low satisfaction with their specialty choice and work-life balance, and had high rates of emotional exhaustion [4]. Additionally, female physicians carry more domestic responsibilities than their male counterparts [5]. A population with added stressors are mid-career female physicians in the military who face unique challenges to include deployments, lack of predictability, biannual Permanent Change of Duty moves, and frequent changes in demands within their career cycles [6].

The Institute for Healthcare Improvement (IHI) developed a robust framework, Improving Joy in Work, which includes nine components that lead to happy, healthy, productive people [7]. Joy in work in turn leads to employee engagement with more productivity and less turnover. This is particularly important in the health care sector given the increasing burnout from the COVID-19 pandemic and concurrent mass exodus of health care workers [8]. The pandemic increased burnout, anxiety, and depression among physicians [9]. One systematic review found that physician burnout prevalence ranged from 6.0 to 99.8% [10]. Risk factors for burnout during the pandemic include female gender, long work hours, and testing positive for COVID-19 [11]. 

Similar to the civilian sector, the United States (U.S.) military also faces physician shortages with added stressors that could lead to burnout [12, 13]. Because mid-career physicians, female physicians, and military physicians have all been identified as potentially vulnerable populations to burnout, this study applied the IHI Improving Joy in Work framework to examine causes of physician burnout among this intersectional group to understand what health system changes need to occur to bring joy, improve morale and resiliency, and mitigate burnout for mid-career female military physicians.

## Methods

We conducted qualitative key informant interviews (KII) of mid-career female military physicians who worked in the Military Health System (MHS) during the COVID-19 pandemic (March 2020 -December 2021). Mid-career was defined as O3-O5 military rank. Using the IHI Improving Joy in Work framework, we developed a semi-structured interview guide. The complete interview guide can be found in Supplemental Appendix 1. We recruited participants using purposive snow-ball sampling through email and conducted semi-structured, in-depth interviews virtually using the Google Meets platform. Written informed consent was waived for this study. Oral consent was obtained prior to each interview.

We conducted virtual interviews from February 1, 2023-March 30, 2023. Two researchers conducted the interviews. One researcher guided the discussion and another took digital notes. Interviews were not recorded. Notes were collated and de-identified to ensure confidentiality. The study team analyzed the notes using deductive thematic content analysis and open coding techniques with the help of Nvivo software version 20. We began with expected themes based on the IHI Framework for Improving Joy in Work and identified any emerging themes while reading through the notes. Notes were then coded and codes were synthesized to derive overarching themes related to the IHI Framework for Improving Joy in Work. This study was found exempt by the Institutional Review Board (IRB) of the Uniformed Services University of the Health Sciences (USUHS) and followed the Standards for Reporting Qualitative Research guidelines.

## Results

We interviewed 22 female mid-career military physicians and reached thematic saturation. Our sample was between the ages of 30 and 44 and worked in the MHS between March 2020-December 2021. The majority of participants were Caucasian, followed by Asian/Pacific Islanders. Half of the participants were in the Army, while the next largest group were in the Navy. The majority of the respondents were Majors in the Navy and Army or Lieutenant Commanders in the Navy. Most respondents were married and 7 out of 22 had children during the pandemic. Respondents were mainly Primary Care physicians in OB-GYN, Pediatrics, Family Practice, Preventive Medicine, and Emergency Medicine with a few Surgery and Surgical subspecialties (Table 1).

**Table 1:** Characteristics of participants

Participant	Branch	Specialty	Participant	Branch	Specialty
Physician 1	Army	Pediatrician	Physician 12	Air Force	Pediatric Subspecialty
Physician 2	Army	Pediatrician	Physician 13	Army	Family Practice
Physician 3	Army	Family Practice	Physician 14	Army	Allergist
Physician 4	Air Force	Preventtive Medicine	Physician 15	Navy	Flight Surgeon
Physician 5	Army	Pediatrician Subspecialty	Physician 16	Navy	Flight Surgeon
Physician 6	Army	Pediatrician	Physician 17	Army	OBGYN
Physician 7	Navy	Preventive Medicine	Physician 18	Army	Surgeon
Physician 8	Army	Family Practice	Physician 19	Army	Anesthesiologist
Physician 9	Air Force	Preventive Medicine	Physician 20	Navy	Family Practice
Physician 10	Army	Emergency Medicine	Physician 21	Air Force	Family Practice
Physician 11	Navy	Anesthesiologist	Physician 22	Navy	Occupational Medicine

The top three reasons why female physicians joined the military were sense of duty, financial reasons, and legacy. All but two participants spontaneously reported feeling burnout themselves and knew others who did as well during the COVID-19 pandemic. The two physicians that did not report burnout served in remote locations during the pandemic. Residency, being stationed at resource-poor locations, and uncertain transitional periods were equally cited as the most stressful time period in their careers.

Eight overarching themes were adapted from the IHI framework for Improving Joy at Work: camaraderie and teamwork, choice and autonomy, meaning and purpose, participative management, physical and psychological safety, recognition and rewards, and wellness and resilience. An additional theme was added by the researchers for the purpose of the study, COVID-19 (Table 2).

**Table 2:** Themes identified through deductive analysis of the IHI framework

Overarching Themes
**Camaraderie and Teamwork**
Importance of building rapport
Good relationships with colleagues
**Choice and Autonomy**
Flexibility in work schedule
Administrative burden
Technology issues impeding work
Telemedicine
**Meaning and Purpose**
Ability to mentor other providers
Ability to teach patients about their health
Make a difference in patients’ lives
Pride of serving in the military
**Participative Management**
Frustrations of not being heard by senior leaders
Not feeling supported by leadership
**Physical and Psychological Safety**
Staffing issues
Long work hours and fatigue
Barriers to seeking mental healthcare
**Recognition and Rewards**
Being acknowledged by patients
Not being recognized by leadership or peers
**Wellness and Resilience**
Ability to have a work life balance
Spend time with family, pets, friends
Ability to take care of physical and nutritional needs
**COVID-19**
Staffing issues
Burnout
Lack of PPE
Mental toll of patient deaths
Easier work schedule
Importance of human connections

### Camaraderie and teamwork

Camaraderie and teamwork are described as social cohesion through productive, trusting relationships [7]. All participants discussed the importance of building rapport and having a good relationship with coworkers. Having a connection with others and the ability to work as a cohesive team led to higher levels of engagement and joy in their work.

*Whenever you get a break, or pass someone in the hallway, or get a laugh– those micro moments of connection make a difference.* Physician 21.

### Choice and autonomy

Participants frequently cited their want for choice and flexibility in their work and their desire for a predictable work schedule whenever possible. Two additional themes emerged from Choice and Autonomy: Administrative Burden and Technology Issues. Participants cited the Administrative Burden as a key factor leading to burnout as well as frequent technical difficulties with the electronic health record (EHR) system.

*Having EHR working. Biggest burden. There were several times where you have Genesis down day and that will just totally ruin your day. Really hard to get anything done, hard to address all patient concerns.* Physician 3.

### Meaning and purpose

The IHI Framework describes the importance of finding meaning in work [7]. Participants talked about how Meaning and Purpose contributed to them having a good day and enjoying their practice. Many talked about their love of mentoring other providers or being able to teach their patients. All participants spoke about their desire to help their patients and make a difference in their lives.

*Cliché but wanting to make it feel as if I made a difference.* Physician 20.

An emerging theme was Military Pride. All but 1 participant spoke about their pride in serving in the military and caring for their patients. The one who was not proud later acknowledged how burnt out she felt.

### Participative management

The IHI framework defines Participative Management as leaders who engage before acting, inform others, and listen [7]. Our participants described the lack of Participative Management in their work and the negative impact from not feeling supported and not feeling heard.

*Frustrations of not being heard by leadership. When we constantly say this is not working and give them ideas. Those ideas aren’t heard and that’s affecting morale. Not feeling heard or not seeing a change happen when you’re advocating for it.* Physician 9.

### Physical and psychological safety

Physical and Psychological Safety is defined as ensuring the people feel free from physical harm in the workplace as well as a hospitable climate where individuals are free to express opinions without reprimand [7]. Participants described how staffing issues contributed to long work hours and fatigue. Participants also spoke about the barriers to obtaining mental health care services. A few of the participants mentioned gender discrimination being a challenge in their daily work.

*Barriers to seeking mental healthcare in the military are extremely high. Leadership may have good intentions but don’t make it accessible. Also, in medicine, it can be challenging when it comes to licensing. It feels punitive getting care.* Physician 10.

### Recognition and rewards

The IHI Framework acknowledges the importance of recognition and celebration of team accomplishments in the workplace [7]. Participants spoke about their appreciation when patients say ‘thank you’ or they are acknowledged for helping others. They also discussed the consequences when themselves or their peers did not feel recognition for their work from leadership.

*I have seen in my specialty mass exoduses of people. I think some would have stuck around longer if they were treated better.* Physician 16.

#### Wellness and resilience

Participants described how they felt like their best selves when they had work-life balance and did not need to choose work over family. Family, friends, and childcare were described as participants greatest support system. Participants talked about the importance of being able to take care of themselves physically by finding time to work out and eat healthy. One participant described how not having work-life balance exacerbated burnout.

*When I’m sleep deprived or when I’m not able to take care of myself like I should.* Physician 20.

### COVID-19

In addition to the factors described in the IHI Framework for Improving Joy in Work, our study asked about the impact of COVID-19 on physician burnout. Participants described the challenges of COVID-19 from the rapidly changing schedules and cancelled deployments to long work hours, staffing issues, lack of PPE, and difficulties in dealing with patient deaths.

*Constant overwork. There was no off. Everyone (physician, nurses, healthcare providers) was on all the time. Leadership was changing decisions based on their opinions and not based on medical expertise.* Physician 9.

Other participants discussed how the COVID-19 pandemic helped them to achieve work life balance due to scheduling constraints.

*Parts of the pandemic that was helpful for me because there was so much time away from work; felt like life actually balanced out; more time to study; more time to eat well and exercise.* Physician 19.

Additionally, participants discussed how the human connection component of work became more important to them after the pandemic.

## Discussion

We interviewed 22 mid-career active duty service women physicians during the Spring of 2023 who provided patient care in the MHS during the COVID-19 pandemic. Our findings were consistent with previous civilian literature as the majority of our respondents reporting burnout were younger, worked long hours, and had lower job satisfaction [14]. Previous studies report that mid-career physicians were the most likely to quit practicing medicine for reasons other than retirement [4]. Our study identified some factors specific to the military population and found that mid-career female military physicians were leaving the military due to inability to balance military operational needs and personal needs. Common previously cited organizational factors that led to physician burnout include leadership behaviors, high workload expectations, insufficient rewards, limited interpersonal collaboration, and limited opportunities for advancement and social support [2]. We confirm that these organizational factors are also predictors of military female physician burnout.

Physician burnout was amplified by the COVID-19 pandemic impacting health system dynamics around patient safety, professionalism, work culture and worker retention [15]. Our study confirms this as all but two physicians reported feeling burnt out and knowing others who were as well. However, our study also showed that for a few individuals, COVID-19 brought work life balance to a reality. Overwhelmingly our study population has experienced burnout which is deemed an occupational hazard if prolonged as it leads to worsened quality of care, increased cost of care, increased physician substance use disorder, poor self-care, depression, suicidal ideation, and suicide [1]. The IHI Framework for Improving Joy in Work allowed us to identify key areas of these individual’s lives where their work environment is contributing to burnout.

Our participants placed importance on camaraderie and teamwork in the workplace with many stating that the best part of their day was interactions with colleagues. This corresponds with a study showing sense of community to be an anchoring factor for joy in work [16]. However, participants also discussed lack of participatory management from leadership which demonstrates lack of teamwork on the part of leadership. Lack of team cohesion can lead to medical errors which are detrimental to patient care and outcomes [17]. Leadership has a key function in promoting teamwork as their contributions can have a direct impact on organizational culture [18]. Participants also discussed the importance of rewards and recognition. Praise for good work is needed and shown to mitigate burnout [19], however, participants felt that they were constantly being told to do more with less and their successes were never recognized. Based on participant responses leadership from all levels within Military Treatment Facilities (MTFs) should focus on servant leadership and listen to grievances from providers to optimize joy in the workplace.

Additionally, participants wanted predictability and flexibility in their schedules and did not want to be bogged down with administrative work and training on top of their caseloads. Training examples include military-specific trainings such a cyber-awareness, suicide awareness, and operations security. General training requirements include annual regulatory training, and Health Insurance Portability and Accountability Act and Privacy Act (HIPAA) trainings. These trainings can be in person or online across multiple platforms. Altogether, active duty resident physicians have an average of 17 h of training annually, 65% of which is military specific which takes time away from patient care [20]. 

As an added stressor, all participants discussed their frustration with the EHR system. The MHS rolled out a new EHR called MHS GENESIS in 2017 [21]. Since the rollout, pilot studies have concluded low usability scores for MHS GENESIS with practitioners wanting to return to legacy systems [22]. EHR’s meant to improve patient care, are a documented source of work dissatisfaction and burnout among clinicians [23, 24]. Potential strategies to improve these stressors are protected time for comprehensive training on new EHR systems prior to launch and administrative assistants to help with last minute administrative tasks. Alternatively, protected time for administrative work and trainings would be beneficial.

Physical and psychological safety and wellness and resilience are key to thriving in the workplace. Participants expressed the stresses of long work hours and understaffing contributing to burnout coupled with the lack of access to mental health care. Many discussed that they felt at their best when they were able to exercise, eat healthy, and engage with their support systems, however, most overall did not feel that they had a work-life balance on a regular basis. These factors are clearly linked in the literature to physician burnout [25]. Lack of access to behavioral health care is particularly concerning as previous research has shown female physicians to lack access in the military [26]. Steps should be taken to address this gap, by creating pathways for female physicians to access mental health services. An emerging theme was gender discrimination felt by female physicians. Female physicians felt that the lack of female physicians in general and in senior roles lead to a culture of a “boys’ club” (Physician 11). They felt that while pay was the same for each gender in the military, they ended up picking up more additional duties and administrative work compared to men. Literature has shown that workplace culture can contribute to gender disparities in terms of burnout [27]. A small proportion of participants spoke about issues with childcare and breastfeeding support, but it was not a resounding theme. Additionally, a few participants discussed issues in obtaining childcare which is concerning given the role of these mothers in national security. Improvements in workplace culture within MTFs are encouraged to promote gender equality.

Almost all participants felt that taking care of people and providing excellent and effective patient care mattered to them and provided meaning and purpose in work. The most meaningful parts of their day were helping patients resolve medical problems, working with their chosen population, mentoring or providing medical education, and making a positive impact. This corresponds with literature describing the importance of connections between providers and patients and how these relationships can promote physician resilience against burnout [28]. MTF leadership should ensure physicians have adequate time to provide quality care for their patients simultaneously ensuring that staff have protected time for required trainings and administrative tasks.

The feedback from mid-career female physicians points to several areas of focus that leaders within the MHS can work on to make immediate impacts. MHS leadership can incorporate more participative management for this demographic by engaging female physicians monthly or having a forum where they can speak up openly about potential improvements. A policy path towards increasing visible female military healthcare leadership positions at military health training installations and schools so service members become accustomed to engaging with female leaders at the beginning of their service time is encouraged. Physical and psychological safety within the MHS can be created through leaders actively demonstrating support for healthcare worker mental healthcare and promoting telehealth mental health appointments for physicians. MHS leaders can develop more female military leadership training courses and consciously promote integration of female leaders in higher ranks across the military organization. Additionally, MHS leadership can focus on improving ancillary benefits for physicians to include cost effective installation childcare programs with adequate capacity, supplemental childcare vouchers, flexible work options, hospital or clinic-based employee family wellness programs, and tailored programs for physician staff.

## Limitations

Respondent’s perspectives are limited to their location and the timeframe during the COVID-19 pandemic, which we designated from March 2020-December 2021. As the interviews were conducted 2 years after the pandemic, responses may be subject to recall bias. There was only a short time to collect the data from the beginning of February to the end of March 2023. Most participants were White mid-career female physicians in their early 30s in the primary care field; thus, this study lacks perspectives of senior physicians, male physicians or individuals working in the surgical field; hence it is not a representative sample of MHS providers. Likewise, our study population is over representative of White women compared to the racial breakdown of active duty service women. Military specific factors may limit generalizability to civilian population. Future studies should focus on expanding these analyses to reach additional study populations negatively impacted by burnout.

## Conclusion

Mid-career female physicians serving in the armed forces experience physician burnout exacerbated by the COVID-19 pandemic. MHS leadership should work to alleviate factors contributing to burnout and maintain adequate providers to care for family members and ensure readiness of the armed forces. More research is needed to include quantitative and qualitative studies for this subpopulation to gain granularity of the workforce behind the overall MHS.

### Electronic supplementary material

Below is the link to the electronic supplementary material.

Supplementary Material 1

## Data Availability

The dataset generated and analyzed during the current study is available from the corresponding author on reasonable request.

## Declarations

### Disclaimer

The contents of this publication are the sole responsibility of the authors and do not necessarily reflect the views, assertions, opinions or policies of the Uniformed Services University of the Health Sciences (USUHS), the Henry M. Jackson Foundation for the Advancement of Military Medicine, Inc. (HJF), the Department of Defense (DoD), or the Departments of the Army, Navy, or Air Force. Mention of trade names, commercial products, or organizations does not imply endorsement by the U.S. Government.
