# Supplementary material for: Assessing mid-career female physician burnout in the military health system: finding joy in practice after the COVID-19 pandemic
Source: BMC Public Health. 2024 Mar 20;24:862. doi: 10.1186/s12889-024-18357-5 (PMC10953201; doi:10.1186/s12889-024-18357-5)
Supplement: Supplementary file 1 — Supplementary Material 1 [file 12889_2024_18357_MOESM1_ESM.docx]

**CHSR: Assessing Mid-Career Female Physician Burnout in the Military Health System: How to find joy in practice after the COVID-19 Pandemic**

**Participant: 0**

**Date: month, year**

**Researchers: names of interviewer and notetaker**

**Introduction**

The CHSR is conducting a study of physician burnout and mitigation strategies among mid-career female physicians during the COVID-19 pandemic. You are one of several volunteers that we have asked to share their perspectives. Your experience will be used to provide insight on how the MHS supports the mental wellbeing of its female physicians.

**What will we be doing?**

First, we ask you some background questions. Then we will ask you about your feelings and experiences working as a physician during the COVID-19 pandemic. There are no right or wrong answers, we just want to learn more about your experience. You are free to share with us anything that you experienced firsthand or anything you heard from others. We are not compelled to disclose information about subjects as we are not mandated reporters. However, please do not disclose any information in the interview if it will put you or others at risk. We are happy to help engage you with mental health services if you feel distressed during or after this interview.

**Who is doing what?**

I will be leading the interview, xxxx will be taking notes.

Do you have any questions for us before we get started?

**Demographics**

| 1 | What is your current age? |
| --- | --- |
|  | |

| 2 | What race or races do you identify as? |
| --- | --- |
|  | |

| 3 | What is your current rank and branch of service? |
| --- | --- |
|  | |

| 4 | Were you a physician or surgeon during the pandemic, March 2020-December 2021? |
| --- | --- |
|  | |

| 5 | What was your marital status during the pandemic, March 2020-December 2021? |
| --- | --- |
|  | |

| 6 | Did you have children during the pandemic? |
| --- | --- |
|  | |

| 6A | (If they have children) Were any of your children under 5 years old? |
| --- | --- |
|  | |

**Background Probing Questions**

| 7 | How did you decide to work in military health care? |
| --- | --- |
|  | |

| 8 | How did you choose your branch and specialty? |
| --- | --- |
|  | |

**Community Assessment Probing Questions**

| 9 | Do you know of any physician colleagues who have felt burnt out during the pandemic? What was the situation? |
| --- | --- |
|  | |

| 10 | What do you think contributes to other military physicians’ burnout? |
| --- | --- |
|  | |

**Values-Based Self-Assessment Probing Questions**

| 11 | What matters to you in your daily work? During the pandemic? and now? |
| --- | --- |
|  | |

| 12 | What helps you have a good day? During the pandemic? and now? |
| --- | --- |
|  | |

| 13 | What is the most meaningful part of your work? During the pandemic? and now? |
| --- | --- |
|  | |

| 14 | What is the best part of your work? During the pandemic? and now? |
| --- | --- |
|  | |

| 15 | What are some of the greatest sources of support for you? (Social, mental, physical, child care, etc.) During the pandemic? and now? |
| --- | --- |
|  | |

| 16 | What time periods during your military career did you find to be the most stressful? |
| --- | --- |
|  | |

| 17 | What makes you proud to work with the military? |
| --- | --- |
|  | |

| 18 | When you are at your best, what does that look like? |
| --- | --- |
|  | |

**Impediment Assessment Probing Questions**

| 19 | What gets in the way of a good day? During the pandemic? and now? |
| --- | --- |
|  | |

| 20 | What frustrates you in your day? During the pandemic? and now? |
| --- | --- |
|  | |

| 21 | What are some barriers for you to finding joy in the workplace? During the pandemic? and now? |
| --- | --- |
|  | |

**Organizational Work System Improvement Recommendations Probing Questions**

| 22 | If you could change anything about the Military Health System, what would that be and why? |
| --- | --- |
|  | |

| 23 | What else would you want the Military Health System leadership to know regarding your life or career concerns? |
| --- | --- |
|  | |
